# Improved long-term survival with home hemodialysis compared with institutional hemodialysis and peritoneal dialysis: a matched cohort study

**DOI:** 10.1186/s12882-019-1245-x

**Published:** 2019-02-13

**Authors:** Helena Rydell, Kerstin Ivarsson, Martin Almquist, Mårten Segelmark, Naomi Clyne

**Affiliations:** 1 Department of Clinical Sciences Lund, University, Skane University Hospital, Nephrology Lund, Lund, Sweden; 2 Department of Clinical Sciences Lund, Pediatric psychiatry, Lund University, Skane University Hospital, Lund, Sweden; 3 Department of Clinical Sciences, Lund University, Skane University Hospital Lund Surgery, Lund, Sweden

**Keywords:** Home hemodialysis, Peritoneal dialysis, Institutional hemodialysis, Survival, Renal graft survival

## Abstract

**Background:**

The survival rate for dialysis patients is poor. Previous studies have shown improved survival with home hemodialysis (HHD), but this could be due to patient selection, since HHD patients tend to be younger and healthier. The aim of the present study is to analyse the long-term effects of HHD on patient survival and on subsequent renal transplantation, compared with institutional hemodialysis (IHD) and peritoneal dialysis (PD), taking age and comorbidity into account.

**Methods:**

Patients starting HHD as initial renal replacement therapy (RRT) were matched with patients on IHD or PD, according to gender, age, Charlson Comorbidity Index and start date of RRT, using the Swedish Renal Registry from 1991 to 2012. Survival analyses were performed as intention-to-treat (disregarding changes in RRT) and per-protocol (as on initial RRT).

**Results:**

A total of 152 patients with HHD as initial RRT were matched with 608 IHD and 456 PD patients, respectively. Median survival was longer for HHD in intention-to-treat analyses: 18.5 years compared with 11.9 for IHD (*p* <  0.001) and 15.0 for PD (*p* = 0.002). The difference remained significant in per-protocol analyses omitting the contribution of subsequent transplantation. Patients on HHD were more likely to receive a renal transplant compared with IHD and PD, although treatment modality did not affect subsequent graft survival (*p* > 0.05).

**Conclusion:**

HHD as initial RRT showed improved long-term patient survival compared with IHD and PD. This survival advantage persisted after matching and adjusting for a higher transplantation rate. Dialysis modality had no impact on subsequent graft survival.

## Background

Survival for patients on dialysis is poor, despite improvement over time both in the US and in Europe [1, 2]. In Sweden, the annual mortality rate for patients on dialysis was 28–30% between 1991 and 1999 and decreased to around 20% in 2010, after which it has remained stable [3].

Earlier studies have indicated improved survival for patients on home hemodialysis (HHD) compared with institutional hemodialysis (IHD) [4–10] and peritoneal dialysis (PD) [6, 11–14]. For patients starting HHD at Lund University Hospital, we have previously reported an annual mortality rate of less than 5%, after more than 30 years of follow-up [9]. Most earlier studies have reported short-term survival, five years or less [4, 11, 12, 14], have not followed patients into the twenty-first century nor taken comorbidity into account. As the overall survival for dialysis patients has improved during the twenty-first century, and as patients starting HHD are usually younger and healthier than patients on IHD or PD, the survival advantage for HHD beyond patient selection is still unclear. This study comprises patients starting dialysis between 1991 and 2012 and the matching is performed with a comorbidity index, which is a composite measure for comorbidity.

Patients eligible for HHD are most often also eligible for renal transplantation. There are, to our knowledge, no previous reports studying the effects of dialysis modality on subsequent renal graft survival.

The primary aim of this study was to compare long-term survival for patients with HHD as initial renal replacement therapy (RRT) with matched control patients with either IHD or PD as initial RRT. The secondary aim was to compare subsequent renal graft survival for patients with either HHD, IHD or PD as initial RRT.

## Methods

### Definitions of initial renal replacement therapy

**Fig. 1:**
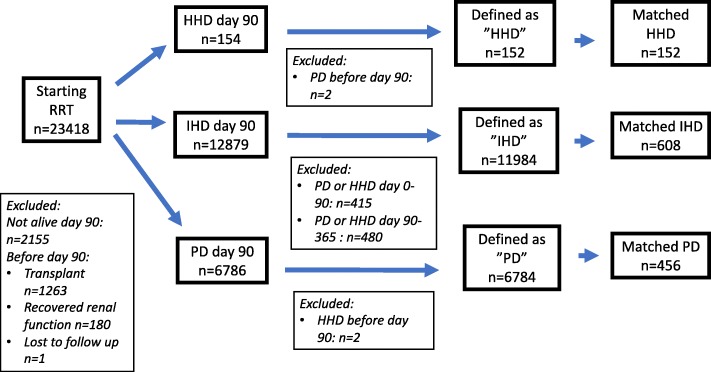
Inclusion and exclusion of matched patients with HHD, IHD and PD as initial renal replacement therapy

HHD, IHD or PD as initial RRT were defined as the modality registered in the Swedish Renal Registry (SRR) at day 90 after start of RRT. A failing renal transplant or a period of recovered renal function before day 90 were exclusion criteria. To be defined as HHD as first RRT, a patient was not allowed to have received PD before day 90. To be defined as PD, a patient was not allowed to have had HHD before day 90. To be defined as IHD, no other RRT was allowed during the first year after start of RRT, except for transplantation after day 90 (Fig. 1).

### Inclusion criteria

All patients 18 years or older, registered in the SRR and starting RRT between January 1st 1991 and December 31st 2012 were eligible for inclusion if they fulfilled the criteria of HHD, IHD or PD as initial RRT.

### Matching

We created two matched control sets by matching HHD patients separately with PD (1:3) and IHD (1:4) patients. Gender, Charlson Comorbidity Index, age (+/− 3 years) and date for start of RRT (+/− 3 years) were used as matching criteria. Matching was performed at day 0 of RRT. Matching was performed in loops starting with the HHD patients with the least possible matches. The loops continued as long as there were controls fulfilling matching criteria for all HHD patients.

Charlson Comorbidity Index [15] was defined using all discharge diagnoses in the Swedish Inpatient Registry until the start date of RRT as previously described [16].

### Data collection

Start dates and the dates of changes in RRT, dates of birth and renal diagnoses were collected from the SRR. Discharge diagnoses and dates of hospital admissions were collected from the Swedish Inpatient Registry. Dates of death were collected from the Swedish Mortality Database.

### Patient survival

Survival was calculated from day 0 of RRT. All patients were followed until death or December 31st 2013. No patient was registered as lost to follow-up in the SRR.

Two different survival analyses were performed. In intention-to-treat analysis (ITT), patients were considered at risk also after switching to other RRT. As renal diagnosis was not considered in the matching, it was included in a bivariable Cox regression ITT survival analysis. A sensitivity analysis was performed with censoring at recovery of native renal function.

In addition, two different per-protocol analyses were performed: “on initial RRT” analysis with censoring for all switches from the group modality and “on dialysis treatment” analysis with censoring at renal transplantation, recovered native renal function and end of study.

### Subsequent renal transplantation

Renal graft survival was studied in all patients who had a subsequent renal transplantation and in a subgroup of patients who had a renal transplantation while still on their initial RRT. As the subgroups with subsequent transplants were not matched, multivariable Cox regression analysis was performed including decade of start of RRT, gender, age and Charlson Comorbidity Index at day 0 for RRT, renal diagnosis, dialysis vintage at the date of transplantation and whether the graft was from a living or deceased donor. Graft failure was defined as the only event in this analysis. Censoring was performed at date of death and at end of study.

### Statistical analysis

Calculations of Charlson Comorbidity Index were performed using STATA software version 12. Definitions of groups and matching were made using SAS. All survival analyses were performed with IBM SPSS version 23.

For both patient and renal graft survival analysis, Kaplan Meier curves were used as well as multivariable Cox regression analyses. Log rank test was used for comparison of patient survival. Descriptive data and survival are given as medians, interquartile ranges (IQR) and means.

## Results

### Patient characteristics

During 1991 to 2012, 152 patients started RRT in Sweden with HHD as their initial treatment modality, according to the criteria used in this study. Their mean age was 50.2 years and 82% were male. A Charlson Comorbidity Index was assigned retrospectively to each patient using hospital discharge diagnoses. Most patients had an index of 0 (63%), 28% had an index of 1, 8% had an index of 2 and 2% an index of 3 (Table 1).

**Table 1:** Patient characteristics at start of renal replacement therapy in a cohort of Swedish HHD patients and two matched control cohorts of IHD and PD patients

	HHD	IHD	PD
Patients number	152	608	456
Year of start percent (n)			
● 1991–1999	52% (79)	48% (290)	48% (221)
● 2000–2009	36% (54)	42% (253)	42% (193)
● 2010–2012	13% (19)	11% (65)	9% (42)
Median age (IQR) *years*	50.2 (42.1–58.2)	50.1 (42.4–58.1)	50.1 (42.2–58.0)
Gender male percent (n)	82% (124)	82% (496)	82% (372)
Charlson index percent (n)			
● 0	63% (95)	63% (380)	63% (285)
● 1	28% (42)	28% (168)	28% (126)
● 2	8% (12)	8% (48)	8% (36)
● 3	2% (3)	2% (12)	2% (9)
Renal diagnosis *percent (n)*			
● Diabetes mellitus	10% (15)	20% (123)	27% (122)
● Glomerulonephritis	30% (46)	25% (149)	28% (126)
● Hypertension	6% (9)	7% (43)	5% (23)
● APCKD^a^	15% (23)	10% (62)	9% (43)
● Pyelonephritis	4% (6)	3% (21)	3% (12)
● Other	28% (43)	23% (138)	20% (89)
● Unspecified	6% (10)	12% (72)	9% (41)

^a^Adult polycystic kidney disease

This cohort of incident HHD patients was compared with two matched cohorts - one starting on IHD and one starting on PD. Matched controls were recruited for each HHD patient, generating an IHD cohort of 608 and a PD cohort of 456 patients. As shown in Table 1, the cohorts were well-matched with respect to age, gender and co-morbidity index.

The most common renal diagnosis was glomerulonephritis in all three groups followed by adult polycystic kidney disease for HHD and diabetic nephropathy for the IHD and PD patients (Table 1).

### Changes in RRT during follow-up

Median follow-up duration was 10.4 years for HHD (IQR 5.9–15.4), 7.0 years for IHD (IQR 2.8–12.8) and 7.5 years for PD patients (IQR 3.4–13.8). According to the definitions of initial RRT, no patient had recovered renal function before day 90. However, after day 90, one HHD patient, 11 IHD and six PD patients had recovered enough renal function for a pause in dialysis treatment.

Most patients changed RRT during the follow-up period, although 25 HHD (16%), 282 IHD (46%) and 74 PD (16%) patients continued on the same dialysis modality after day 90 until death or December 31st 2013. Renal transplantation was the most common RRT subsequent to the initial dialysis modality but switches between dialysis modalities were also common, resulting in several periods on the initial dialysis modality for some patients (Table 2). Among the HHD patients, 9% had more than one period on HHD (1 to 3 periods). Among IHD and PD patients, 13% had more than one period on their initial RRT. Of the HHD and PD patients, 24 and 38% respectively had one to three periods on IHD. However, switches to HHD or PD from other dialysis modalities were uncommon. Only 2% of the IHD and 1% of the PD patients had a period on HHD, and only 12% of the IHD patients and zero HHD patient switched to PD.

**Table 2:** Duration and frequency of initial and subsequent renal replacement therapies for the respective cohorts

	HHD	IHD	PD
Initial RRT			
Median duration (IQR) *years*			
First period with	2.1	2.3	1.4
HHD/IHD/PD	(1.1–3.1; *n* = 152)	(1.1–3.9; *n* = 608)	(0.8–2.4; *n* = 456)
Total treatment with	2.4	2.6	1.5
HHD/IHD/PD	(1.2–3.6; *n* = 152)	(1.3–4.9; *n* = 608)	(0.9–2.7; *n* = 456)
Other RRT			
Median duration (IQR) *years*			
Renal transplantation	8.9	8.6	8.4
	(5.1–13.5; *n* = 114)	(3.8–12.3; *n* = 312)	(4.3–13.1; *n* = 311)
HHD	–	3.2 (2.3–6.8; *n* = 10)	0.8 (0.3–0.8; *n* = 5)
IHD	2.3 (0.6–4.8; *n* = 36)	–	3.0 (0.7–8.8; *n* = 174)
PD	0	1.7 (0.6–2.7; n = 15)	–

### Patient survival

**Fig. 2:**
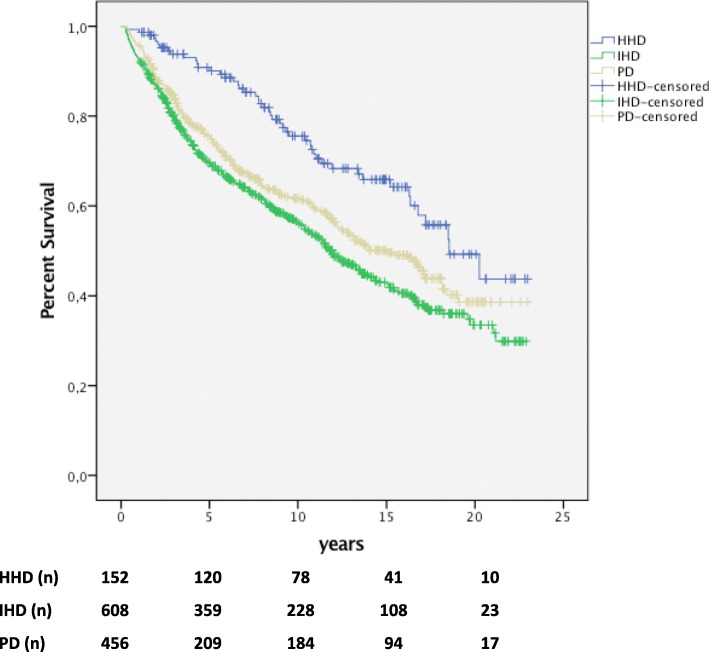
Overall survival. Superior overall survival (intention-to-treat analysis) for incident patients with HHD (*n* = 152) as first renal replacement therapy (RRT) compared with matched patients with IHD (*n* = 608; *p* < 0.001) and PD (*n* = 456; *p* = 0.002) as first RRT. In the analyses, changes to other modalities were not considered and censoring was only performed at the end of the study

The HHD cohort showed a superior overall survival compared with the matched IHD cohort (*p* <  0.001) and the matched PD cohort (*p* = 0.002). In this survival analysis, disregarding all changes in RRT, median survival was 18.5 years (IQR 10.4 - not available) for HHD patients, 11.9 years for IHD patients (IQR 3.8 - not available) and 15.0 years for PD patients (IQR 5.1 – not available). At the end of the study, on December 31st 2013, 104 HHD (68%), 307 IHD (50%) and 256 (56%) PD patients were still alive. Five, ten and twenty years’ survival was respectively 91, 76 and 49% for HHD patients; 70, 57 and 34% for IHD patients; and 76, 62 and 39% for PD patients (Fig. 2, Table 3).

**Table 3:** Survival for HHD patients and matched IHD and PD patients

Overall survival			
With censoring only at end of study			
	HHD	IHD	PD
Median (IQR) *years*	18.5 (10.4-NA^a^)	11.9 (3.8- NA^a^)	15.0 (5.1- NA^a^)
Comparison with HHD (*p* value)	–	< 0.001	0.002
Mean *years*	16.6	12.5	13.8
With censoring at end of study and recovered native renal function			
	HHD	IHD	PD
Median (IQR) *years*	18.5 (10.4- NA^a^)	12.0 (3.8- NA^a^)	15.1 (5.1- NA^a^)
Comparison with HHD (*p* value)	–	< 0.001	0.003
Mean *years*	16.5	12.6	13.9
Survival on initial RRT only			
With censoring at all changes from the initial modality			
	HHD	IHD	PD
Median (IQR) *years*	NA^b^ (8.4-NA)	6.3 (2.9–11.5)	6.1 (3.2–6.6)
Comparison with HHD (*p value*)	**–**	< 0.001	< 0.001
Mean *years*	9.8	8.2	7.3
Survival on dialysis treatment only			
With censoring at renal transplantation, recovered native renal function and end of study			
	HHD	IHD	PD
Median (IQR) *years*	8.4 (6.7–10.7)	6.2 (2.9–11.4)	5.2 (3.1–7.5)
Comparison with HHD (*p value)*	**–**	0.001	< 0.001
Mean *years*	8.1	8.0	6.4

^a^ Not Available, survival > 25% at end of follow up

^b^Not Available, survival > 50% at end of follow up

As renal diagnosis was not included in the matching, bivariable Cox regression analyses were performed. HHD remained a factor associated with a favourable prognosis in these analyses compared with both IHD (*p* <  0.001) and PD (*p* = 0.033). The hazard ratio (HR) was 0.55 (95% confidence interval 0.40–0.75) for HHD in the comparison with IHD and 0.70 (0.51–0.97) in the comparison with PD. In the analysis with HHD and IHD, adult polycystic kidney disease (HR 0.38; 95% confidence interval 0.22–0.64; p <  0.001) and glomerulonephritis (HR 0.53; 95% confidence interval 0.35–0.78; *p* = 0.001) were related to an improved prognosis. This was in contrast to diabetes, which was related to a worse prognosis (HR 1.94; 95% confidence interval 1.33–2.84; *p* = 0.001). In the analysis of HHD versus PD, only diabetes was significantly related to survival, showing a worse prognosis (HR = 2.57; 95% confidence interval 1.54–4.31; *p* <  0.000).

**Fig. 3:**
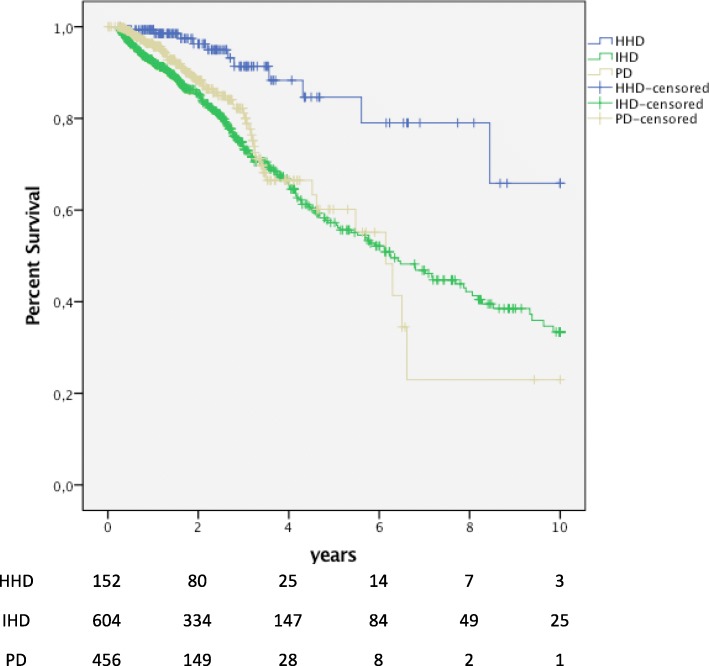
Survival on initial RRT. Superior survival during the first renal replacement therapy (RRT) for patients with HHD (n = 152) compared with matched patients with IHD (n = 608; p < 0.001) and PD (n = 456; p = < 0.001). In these analyses, censoring was performed at all changes to other modalities

Survival analyses per-protocol, with censoring at all switches from the initial RRT, were also performed to remove the contribution of subsequent transplantation and subsequent different dialysis modalities. In these on initial RRT analyses, there were also significant survival advantages for HHD patients in comparison both with IHD (*p* <  0.001) and with PD (*p* < 0.001). Survival was 66% for HHD patients at the end of study on December 31st 2013 (25% quartile 8.4 years; median and 75% quartile not available). Median survival was 6.3 years (IQR 2.9–11.5) for IHD and 6.1 years (IQR 3.2–6.6) for PD patients. The “on initial RRT” five years’ survival was 85% for HHD, 57% for IHD and 60% for PD patients. At 10 years, only three HHD, 25 IHD and one PD patient were still at risk. In survival analysis “on dialysis treatment”, with censoring only at the dates of renal transplantation and lost to follow-up, HHD patients also had significantly longer survival in comparison with IHD (*p* = 0.001) and PD (*p* < 0.001) patients (Fig. 3, Table 3).

### Subsequent renal transplantation

Subsequent renal transplantation was more common among patients with HHD compared with the other modalities. During follow-up, 75% of the HHD patients, 68% of the PD and 51% of the IHD patients received between one and three renal transplants (Table 2).

There was no difference in subsequent renal graft survival between the modalities. Ten years’ graft survival was 75% for HHD (*n* = 114), 72% for IHD (*n* = 313) and 75% for PD (*n* = 311) patients, both when analysed for all patients with a subsequent renal transplantation and for the subgroups with a transplantation immediately subsequent to their initial RRT (HHD *n* = 107, IHD *n* = 304, PD *n* = 219).

In multivariable Cox regression analyses, there were no significant differences in graft survival after HHD and IHD, neither in the analyses for all patients (*p* = 0.416) nor for the subgroups of patients with renal transplantations immediately subsequent to the initial RRT (*p* = 0.489); nor were there significant differences between HHD and PD in the comparisons with all patients (*p* = 0.616) or the subgroups with an immediate subsequent transplantation (*p* = 0.297).

Most other variables in the analyses were not significantly related to survival. However, in both comparisons between HHD and IHD, comprising all patients and those with a renal transplantation immediately subsequent to the initial RRT (*p* = 0.005; *p* = 0.006) and in the comparison between all HHD and all PD patients (*p* = 0.038), an earlier decade of start of RRT was significantly related to a worse graft survival.

## Discussion

This population-based study including all Swedish patients with HHD as initial RRT showed an improved long-term survival for HHD compared with both IHD and PD. We have previously reported similar long-term results in a single-centre population from Skåne University Hospital, for patients starting dialysis between 1983 and 2002, with a five-year survival of 98% for HHD and 71% for IHD patients [8]. Our results are in accordance with most earlier studies [4–8, 10–14], albeit that the latter have generally had shorter follow-up periods, especially those including patients during the twenty-first century only [4, 12, 14]. There are two studies comparing HHD with IHD, one from New Zealand and one from Australia, which included patients starting RRT during the twenty-first century and which followed them for more than five years [7, 10].

In the present study, the hazard ratio of death was 0.55 (*p* < 0.001) for incident HHD patients in comparison with IHD patients and 0.70 (*p* = 0.033) in comparison with PD patients, using intention-to-treat analysis. The magnitude of the survival advantage reported for HHD patients in other studies is affected by different definitions of dialysis modalities, inclusion criteria and methods. One study that defined patients living and dialysing in a long-term care facility as HHD patients showed no survival advantage for HHD compared with IHD or PD. [17] Differences in inclusion criteria, in which exclusively incident or both incident and prevalent patients were accepted, can also have an impact on the results, especially in comparison with PD. A follow-up according to intention-to-treat or per-protocol is another methodological factor behind differences between studies. With follow-up according to per-protocol, the results are not only influenced by the initial RRT but also by the patients who change RRT and who no longer are followed, i.e. those with either an especially good or bad prognosis.

Nevertheless, since more HHD patients than IHD or PD patients received a renal transplant during follow-up, we also conducted survival analyses per-protocol, with censoring at the dates of transplantation. We found that HHD patients still had a survival advantage compared with IHD and PD patients in these analyses, even though the positive impact on survival of subsequent transplantation was omitted.

Probably the most important explanation for the survival advantage for HHD reported in our study, as in most other studies, is a higher dialysis dose, one of the characteristics of HHD. In our previous single-centre study, median weekly dialysis duration was 15.5 for HHD and 12 h for IHD patients [8]. Several studies have reported improved survival related to increased hemodialysis dose, in the form of both longer dialysis sessions and higher weekly frequency [18–27]. As residual renal function declines, the PD dose often becomes insufficient. The smallest advantage for HHD compared with IHD or PD was reported in studies from the US [4, 12, 14, 17]. In one of these studies, a subgroup analysis including only incident patients showed no advantage for HHD in relation to PD. [12] Residual renal function and initial GFR at start of dialysis may also have an impact. Even taking into account differences between countries concerning selection of patients to different modalities and GFR at start of dialysis, dialysis praxis and dialysis dose most likely have a major impact. One such difference in dialysis praxis between the US and Sweden is the use of a low-dialysate-flow dialysis device. This device was not approved in Sweden until 2010 and is still not very common. In Sweden, HHD is usually performed with devices similar to those used in IHD with the capacity to provide high-flow dialysate and long duration, such as nocturnal dialysis.

Extensive patient education is another characteristic of HHD, which also has been shown to improve survival through enhanced patient compliance [28–30]. This is probably an important contributing factor to the difference in survival as compared to IHD, but also to some extent in relation to PD as the patient education required to manage PD is less extensive than with HHD.

Thus, both increased dialysis dose [24, 31–33] and more extensive patient education [34–37] have positive effects on significant prognostic factors which impact cardiovascular mortality, the primary cause of death in dialysis patients.

Patient education might also affect compliance with immunosuppression, resulting in longer graft survival. However, there was no difference in renal graft survival for patients starting on HHD compared with patients starting on PD or IHD. To our knowledge, comparisons of renal graft survival between HHD and IHD or PD as pretransplantation modalities have not been previously reported. However, two earlier studies [38, 39] have reported a similar decline in GFR after renal transplantation subsequent to HHD or IHD.

Because of its retrospective design, there are limitations to this study. The most important is that despite matching, there is still a risk of residual confounding, for instance by socioeconomic factors and smoking. However, the effect of socioeconomic factors probably differs less between the modalities in Sweden than in some other countries, as healthcare is publicly funded and universal; the praxis and access to different RRT is relatively homogenous for the whole population. There were differences in the proportions of renal diagnoses between the groups. However, this was addressed both through matching according to Charlson comorbidity index and through statistical adjustment. As this was a registry study, we did not have information about GFR and other prognostic laboratory measures at start of dialysis, dialysis prescriptions and accesses, which would have been useful. The higher rate of renal transplantations for patients starting with HHD could be a consequence of residual differences in health at start of dialysis, but it could also be caused by a better preservation of the of the health by the dialysis modality. To dissect these two possibilities, a more detailed study focusing on morbidity and hospitalizations would be necessary.

Nonetheless, the study has several merits. The matching was strict and valid albeit that registry data was used to create the comorbidity score. The completeness of the diagnoses in the Swedish Inpatient Registry are more than 99% and their validity has a positive predictive value of 85–95% [40]. As Sweden has a long tradition of high-quality registries, it was possible to follow patients over three decades. Moreover, because of a coverage of nearly 100% in all three registries [40, 41], all Swedish patients starting HHD as initial RRT during the study period could be included.

## Conclusion

This study showed a significant long-term survival advantage for patients starting HHD as initial RRT compared with IHD and PD. Subsequent renal transplantation was more common among patients starting HHD, but there was no difference in subsequent renal graft survival between HHD and IHD or PD as initial RRT. In most countries, patients treated with HHD still comprise less than 5% of the entire dialysis population [1]. The results of this study should encourage increased use of HHD in order to improve the long-term prognosis for dialysis patients.

## Abbreviations

GFR: Glomerular filtration rate
HHD: Home hemodialysis
IHD: Institutional hemodialysis
PD: Peritoneal dialysis
RRT: Renal replacement therapy
SRR: Swedish Renl Registry
